# Clinical profile, imaging characteristics, and operative outcomes of pediatric cystic echinococcosis in Northern Jordan: a single-center retrospective study

**DOI:** 10.3389/fsurg.2026.1779069

**Published:** 2026-04-29

**Authors:** Mohammad Aladaileh, Saleh A. Ba-Shammakh, Joud Al Ramadneh, Yousef Badran, Musab Al-A’athal, Almu’atasim Khamees, Tayseer Al-tawarah, Anas Aljaiuossi, Bassem Al Bataineh, Mohammad AL-Smirat

**Affiliations:** 1 Department of General Surgery and Anesthesia, Faculty of Medicine, Yarmouk University, Irbid, Jordan; 2 Department of General Surgery, Ministry of Health, Amman, Jordan; 3 Faculty of Medicine, Jordan University of Science and Technology, Irbid, Jordan; 4 Department of Public Health and Community Medicine, Jordan University of Science and Technology, Irbid, Jordan; 5 Department of General Surgery, Urology and Anesthesia, Faculty of Medicine, The Hashemite University, Zarqa, Jordan; 6 Department of Pediatric Surgery, Ministry of Health, Amman, Jordan

**Keywords:** albendazole, echinococcus granulosus, Jordan, open surgery, pediatric hydatid cyst

## Abstract

**Background:**

Cystic echinococcosis is a zoonotic parasitic disease with heterogeneous presentation and stage-conscious management. Data describing the clinical profile, imaging pathways, management patterns, and outcomes of pediatric cases in Jordan remain limited.

**Methods:**

We retrospectively reviewed 41 patients (0–13 years) with radiologically confirmed hydatid cysts treated between March 2022 and May 2025. Diagnosis was established using abdominal ultrasound and/or computed tomography (CT), and cysts were staged according to the Gharbi and WHO criteria. Forty patients underwent open cyst deroofing combined with albendazole (15–20 mg/kg/day; one preoperative course and six months post-operatively), whereas one received medical therapy alone. Collected variables included demographics, exposure history, cyst location and stage, operative metrics (duration and blood loss), length of stay, postoperative complications, and recurrence during follow-up (median 12 months).

**Results:**

The mean age was 9.3 ± 3.1 years; 56% were female, and 76% reported domestic animal contact. Liver involvement was most common (56%), followed by lung (39%); 90% had single-organ disease. Type III cysts predominated (37%). The mean operative time was 105 ± 24 min, and the mean hospital stay was 6 ± 3 days. Postoperative complications occurred in 34% (pneumonia 15% and intra-abdominal collections 10%). Recurrence during follow-up (median 12 months) was 4.9%.No demographic or exposure factors significantly predicted complications or recurrence (*p* > 0.05).

**Conclusions:**

Open deroofing plus albendazole in Northern Jordanian children was associated with low recurrence and acceptable morbidity. Expanded ultrasound screening, community zoonosis education, and WHO-aligned protocols are needed.

## Introduction

Cystic echinococcosis (CE), or hydatid cyst disease, is a parasitic disease caused by the tapeworm *Echinococcus granulosus*. Cystic echinococcosis results from cysts formed by the larval stage of the parasite. These cysts develop brood capsules, each containing 30–40 protoscoleces. Each protoscolex is capable of developing into a single adult tapeworm within the host. Cystic echinococcosis is mainly a hepatic and pulmonary disease, producing fluid-filled hydatid cysts in the liver and lungs. Infrequent sites include muscle (5%), bone (3%), kidney (2%), and the spleen, central nervous system, pancreas, and heart (each ∼1%) (1). The disease is endemic in rural livestock communities where sheep and dogs are commonly raised, owing to close contact among humans, sheep, and dogs. It is particularly prevalent in Mediterranean countries, including the Middle East and Turkey, as well as Africa, South America, New Zealand, Australia, Central Asia, and the Russian Federation (2).

Cystic echinococcosis can affect individuals of all age groups; however, pediatric susceptibility may be higher due to environmental exposure, as children are more likely to contact contaminated soil and domestic animals during play, and because an immature immune system may increase vulnerability to infection. The clinical presentation of a hydatid cyst depends on its location and size, and on whether the cyst is intact or ruptures (3). In most cases, asymptomatic or intact hydatid cysts are detected incidentally on imaging; however, larger cysts may cause symptoms due to compression of adjacent organs (3). As mentioned earlier, hydatid cysts primarily affect the liver or lungs. When the liver is involved, presentation is often asymptomatic in the early stages; however, symptoms may include upper right quadrant abdominal pain, abdominal distention, nausea, and vomiting as the cyst enlarges. When the lungs are involved, children may present with chronic cough, hemoptysis, chest pain, and dyspnea; in some cases, pulmonary disease may remain asymptomatic (4–7). Some children may also present with atypical symptoms depending on cyst location: cardiac involvement can lead to palpitations and arrhythmias, bone involvement can result in localized pain and swelling, and central nervous system involvement may present with headache and vomiting. Early diagnosis and management are important to prevent complications, such as rupture and secondary bacterial infection (4–7).

Current WHO and WHO-IWGE guidance emphasizes imaging-based, stage-specific management, with ultrasonography as the preferred first-line modality in organs accessible to ultrasound and CT/MRI used as complementary tools in selected cases. However, real-world practice may differ according to the organ involved, referral patterns, and available expertise (8–11). As previously stated, the disease is endemic in rural and livestock areas where sheep and dog husbandry are common, making it a significant public health issue in Jordan and the Middle East. However, data on pediatric hydatid cyst cases in Jordan remain limited. Therefore, we conducted this study to analyze the clinical characteristics, diagnostic tools, management strategies, and outcomes of pediatric hydatid disease in Northern Jordan.

## Methods

### Participants and settings

This retrospective, single-center study was conducted at Princess Rahma Teaching Hospital, affiliated with Yarmouk University. We reviewed the medical records of children aged 0–13 years with a diagnosis of hydatid cyst disease between March 2022 and May 2025. A total of 41 patients with radiologically confirmed hydatid cysts were included; cases with incomplete documentation or insufficient follow-up were excluded. These represented all eligible pediatric cases managed at our center during the study period, reflecting the experience of a major pediatric referral hospital in Northern Jordan.

### Study design and procedure

Diagnosis was confirmed using computed tomography (CT) and/or ultrasound (US). Data collection encompassed demographic characteristics (age, gender, residency, exposure to animals), clinical symptoms, laboratory results, imaging findings, cyst characteristics, management strategies (medical or surgical), operative details, postoperative complications, hospital stay duration, and recurrence rates. Cyst morphology was described using available imaging records. In abdominal/hepatic lesions where ultrasound-based staging was applicable, cysts were categorized according to the Gharbi and WHO Informal Working Group on Echinococcosis (WHO-IWGE) frameworks. Because this was a retrospective cohort reflecting routine clinical practice, imaging pathways were not standardized, and computed tomography was frequently used for diagnostic clarification, pulmonary disease assessment, multiorgan evaluation, and preoperative planning (8–11) (Tables 1, 2).

**Table 1:** Classification for hydatid cyst type.

Gharbi's 1981	WHO classification	
Type I	CE1	Active
Type II	CE3a	
Type III	CE2	Transition
	CE3b	
Type IV	CE4	Inactive
Type V	CE5	

**Table 2:** Hydatid cyst types according to Gharbi's classification and WHO staging.

Gharbi's	WHO	Number (%)	
Type I	CE1	8 (19.5%)	
Type II	CE3a	4 (9.8%)	
Type III	CE2	6 (14.6%)	15 (36.6%)
	CE3b	9 (22.0%)	
Type IV	CE4	8 (19.5%)	
Type V	CE5	6 (14.6%)	

Surgical management primarily involved open deroofing, with all patients receiving standard perioperative care, including albendazole therapy. Patients were administered albendazole at a dosage of 15–20 mg/kg/day (maximum 800 mg/day),with one course given preoperatively and continuation for six months postoperatively. Treatment decisions were made by the treating surgical team on a case-by-case basis according to clinical presentation, organ involved, symptom burden, imaging findings, complication profile, and operative feasibility. Because the study cohort was derived from a pediatric surgical service, it was enriched for patients referred for operative management. Inactive stage alone should not be interpreted as a routine indication for surgery in all settings (8, 10).

Postoperative follow-up was conducted using CT, US, or both to monitor for recurrence. When US suggested a potential cyst recurrence or was equivocal, confirmation was obtained through CT imaging to ensure accurate diagnosis and further management. Serological testing for echinococcosis was not uniformly available in this retrospective cohort and was therefore used as an adjunct to imaging and clinical assessment rather than as a standalone diagnostic criterion (12).

### Surgical technique

Patients eligible for surgery underwent a thorough preoperative assessment, including imaging studies to characterize the cysts. An open approach was employed to access and identify the cyst. To minimize the risk of dissemination in case of spillage, gauze soaked with 0.5% cetrimide was packed around the cyst. The cyst was then aspirated to collapse, followed by the injection of 0.5% cetrimide, which was left in place for 10 min before complete aspiration. This maneuver was repeated twice. This scolicidal protocol (0.5% cetrimide; 10-minute dwell time; repeated twice) reflects the institution's standard operative practice to reduce spillage-related dissemination. The puncture in the cyst was subsequently widened to facilitate careful extraction of the germinal membrane and daughter cysts. The cyst wall was excised, and the inner epithelial lining was cauterized using monopolar diathermy. The excised specimen was sent for histopathological examination. Hemostasis was ensured, and appropriate suction was performed before placing a 10–14 Fr Redivac drain near the cyst site.

For pulmonary hydatid cysts, lung-preserving open surgery was performed with evacuation of cyst contents and management of the residual cavity according to intraoperative findings, including capitonnage when appropriate, followed by chest tube drainage as indicated (13, 14).For pulmonary hydatid cysts, a chest tube was inserted postoperatively to facilitate fluid drainage and manage potential complications such as postoperative pneumothorax. In cases involving hepatic and renal hydatid cysts, a Redivac drain was placed near the cyst to manage fluid collection and prevent complications.

### Outcomes measured

Short-term postoperative complications included biliary leaks, wound infections, intra-abdominal collections, pneumothorax, pleural effusions, and pneumonia. Long-term outcomes were assessed based on recurrence, which was evaluated using CT and US during follow-up visits.

### Statistical analysis

Continuous variables were summarized as mean ± standard deviation or median (interquartile range), as appropriate. Categorical variables were summarized as frequencies and percentages. Group comparisons were exploratory and performed using the chi-square test or Fisher's exact test for categorical variables, depending on expected cell counts, and the independent-samples t test or Mann–Whitney *U*-test for continuous variables, according to distributional assumptions. A two-sided *p* value < 0.05 was considered statistically significant. Analyses were performed using SPSS version 25 (IBM Corp., Armonk, NY, USA).

### Ethical considerations

This study was approved by the Institutional Review Board (IRB/2024/184) of Yarmouk University, and all procedures adhered to ethical guidelines concerning patient confidentiality and informed consent.

## Results

A total of 41 pediatric patients diagnosed with hydatid cyst disease were included in this study. The majority were female (56.1%, *n* = 23), with the predominant age group being 7–13 years (73.2%, *n* = 30).The vast majority of patients were local residents (78.0%, *n* = 32), and a considerable proportion (75.6%, *n* = 31) reported exposure to domestic animals, primarily dogs (Table 3).

**Table 3:** Baseline patient characteristics, clinical presentation, imaging findings, management, and outcomes (*n* = 41).

Patient characteristics	(*n* = 41)
Age	
0–6 years [*n* (%)]	11 (26.8%)
7–13 years [*n* (%)]	30 (73.2%)
Gender	
Male [*n* (%)] F	18 (43.9%)
Female [*n* (%)]	23 (56.1%)
Residency	
Local [*n* (%)]	32 (78%)
Immigrant [*n* (%)]	9 (22%)
Contact with dogs or domestic animal	
Yes [*n* (%)]	31 (75.6%)
No [*n* (%)]	10 (24.4%)
Clinical symptoms	
Asymptomatic [*n* (%)]	7 (17.1%)
Skin rash [*n* (%)]	2 (4.9%)
Cough [*n* (%)]	13 (31.7%)
Shortness of breath [*n* (%)]	6 (14.6%)
Fever [*n* (%)]	6 (14.6%)
Abdominal pain [*n* (%)]	15 (36.6%)
Anaphylactic shock and rupture [*n* (%)]	2 (4.9%)
Jaundice [*n* (%)]	2 (4.9%)
Laboratory Results	
Unremarkable [*n* (%)]	11 (26.8%)
Leukocytosis [*n* (%)]	13 (31.7%)
Hyper eosinophilia > (0–0.8) x103 [*n* (%)]	13 (31.7%)
Serology (echinococcosis titer)	
Positive [*n* (%)]	10 (24.4%)
Negative [*n* (%)]	11 (26.8%)
Not performed [*n* (%)]	20 (48.8%)
Presentation	
Single organ involvement [*n* (%)]	37 (90.2%)
Two organs involvement [*n* (%)]	2 (4.9%)
Multi-organ [*n* (%)]	2 (4.9%)
Imaging	
CT [*n* (%)]	26 (63.4%)
US [*n* (%)]	0 (0.0%)
Both [*n* (%)]	15 (36.6%)
Locations (single organ)	
Lung [*n* (%)]	16 (39%)
Spleen [*n* (%)]	7 (17.1%)
Liver [*n* (%)]	23 (56.1%)
Kidney [*n* (%)]	1 (2.4%)
Multiple Organ Involvement	
Yes [*n* (%)]	4 (9.8%)
No [*n* (%)]	37 (90.2%)
Number of cysts	
1 [*n* (%)]	24 (58.5%)
2 [*n* (%)]	13 (31.7%)
3 [*n* (%)]	2 (4.9%)
4 [*n* (%)]	2 (4.9%)
Management Strategy	
Invasive approach [*n* (%)]	40 (97.6%)
Medical [*n* (%)]	1 (2.40%)
Type of Surgery	
Open approach [*n* (%)]	40 (97.6%)
Deroofing	40 (97.6%)
Operation	
Operative time [Minutes ± SD]	104.63 ± 23.832
EBL [ml ± SD]	53.66 ± 23.426
LOS [Days ± SD]	6.02 ± 2.697
Timing of Drain removal after surgery [Days ± SD]	4.32 ± 2.196
Post-op complication	
Yes [*n* (%)]	14 (34.1%)
Type of Post-op complication	
Biliary Leak [*n* (%)]	2 (4.9%)
Wound infection [*n* (%)]	2 (4.9%)
Intra-abdominal collection [*n* (%)]	4 (9.8%)
Pneumothorax or pleural effusion [*n* (%)]	4 (9.8%)
Air leak [*n* (%)]	4 (9.8%)
Pneumonia [*n* (%)]	6 (14.6%)
Readmission [*n* (%)]	4 (9.8%)
Follow-up imaging	
CT [*n* (%)]	35 (85.4%)
US [*n* (%)]	2 (4.9%)
Both [*n* (%)]	4 (9.8%)
Recurrence	
Yes [*n* (%)]	2 (4.9%)
Follow-up duration (months)	
12 [*n* (%)]	36 (87.8%)
6 [*n* (%)]	5 (12.2%)

CT, computed tomography; US, ultrasound; n, number.

Laboratory findings are not mutually exclusive (patients may have >1 abnormality).

The most frequently reported clinical presentations included abdominal pain (36.6%, *n* = 15), cough (31.7%, *n* = 13), and fever (14.6%, *n* = 6). Additional symptoms observed were shortness of breath (14.6%, *n* = 6), asymptomatic cases (17.1%, *n* = 7), skin rash (4.9%, *n* = 2), anaphylactic shock with rupture (4.9%, *n* = 2), and jaundice (4.9%, *n* = 2) (Table 3). Laboratory findings revealed that leukocytosis (31.7%, *n* = 13) and hypereosinophilia (31.7%, *n* = 13) were frequently encountered. Serological testing for echinococcosis was positive in 24.4% (*n* = 10) and negative in 26.8% (*n* = 11), while it was not performed in 48.8% (*n* = 20). In this cohort, serology was used as an adjunct to imaging and clinical assessment rather than as a uniform diagnostic requirement (Table 3).

Imaging modalities used in diagnosis varied, with CT being the sole imaging tool in 63.4% (*n* = 26) of cases, while 36.6% (*n* = 15) underwent a combination of US and CT. The liver was the most commonly affected organ (56.1%, *n* = 23), followed by the lungs (39.0%, *n* = 16), spleen (17.1%, *n* = 7), and kidney (2.4%, *n* = 1). Single-organ involvement was predominant (90.2%, *n* = 37), while 4.9% (*n* = 2) of cases exhibited two-organ involvement, and another 4.9% (*n* = 2) presented with multi-organ disease (Table 3). The number of cysts varied among patients, with 58.5% (*n* = 24) having a single cyst, 31.7% (*n* = 13) presenting with two cysts, and a minority presenting with three (4.9%, *n* = 2) or four (4.9%, *n* = 2) cysts (Table 3).

According to Gharbi's classification and WHO staging, type III cysts were the most prevalent (36.6%, *n* = 15), followed by type I (19.5%, *n* = 8) and type IV (19.5%, *n* = 8) (Tables 1, 2). The treatment strategy was predominantly surgical, with open deroofing performed in 97.6% (*n* = 40) of cases, while only one patient (2.4%) underwent medical management. The mean operative time was 104.63 ± 23.83 min, with an estimated blood loss (EBL) of 53.66 ± 23.43 mL. The mean hospital stay was 6.02 ± 2.70 days, and the mean drain removal time was 4.32 ± 2.20 days postoperatively (Table 3).

Postoperative complications were documented in 34.1% (*n* = 14) of cases. Among the most common complications, pneumonia was observed in 14.6% (*n* = 6), while intra-abdominal collections (9.8%, *n* = 4), pneumothorax or pleural effusion (9.8%, *n* = 4), air leaks (9.8%, *n* = 4), and readmission (9.8%, *n* = 4) were also noted. Biliary leak (4.9%, *n* = 2) and wound infection (4.9%, *n* = 2) were the least frequently reported complications (Table 3). The recurrence rate over a median follow-up period of 12 months was 4.9% (*n* = 2) (Table 3).

Statistical analyses indicated no significant associations between age, gender, residency status, or animal exposure and clinical outcomes, including recurrence (*p* = 1.0), postoperative complications (*p* = 0.196–0.719), operative time (*p* = 0.108–0.956), estimated blood loss (*p* = 0.121–0.602), and length of hospital stay (*p* = 0.324–0.869) (Tables 4–11).

**Table 4:** Age group and categorical outcomes: univariable analysis.

Age vs. categorical variables (univariable analysis)	0–6 years (*n* = 11)	7–13 years (*n* = 30)	*P*-value
Asymptomatic [*n* (%)]	1 (9.09)	6 (20)	.651
Multiple Organ Involvement [*n* (%)]	0 (0.0)	4 (13.3)	.559
Post-op complication [*n* (%)]	3 (27.3)	11 (36.6)	.719
Recurrence [*n* (%)]	0 (0.0(	2 (6.7)	1.0

**Table 5:** Age group and continuous outcomes: univariable analysis.

Age vs. continuous variables (univariable analysis)	0–6 years (*n* = 11)	7–13 years (*n* = 30)	*P*-value
Operative time [minutes ± SD]	98.2 ± 14	107 ± 26.3	.3
EBL [mL ± SD]	50 ± 0	55 ± 27.4	.552
LOS [days ± SD]	5.8 ± 1.5	6.1 ± 3.1	.771

EBL, estimated blood loss; LOS, length of stay.

**Table 6:** Sex and categorical outcomes: univariable analysis.

Gender vs. categorical variables (univariable analysis)	Male (*n* = 18)	Female (*n* = 23)	*P*-value
Asymptomatic [*n* (%)]	2 (11.1)	5 (21.7)	.438
Multiple Organ Involvement [*n* (%)]	3 (16.7)	1 (4.35)	.303
Post-op complication [*n* (%)]	4 (22.2)	10 (43.5)	.196
Recurrence [*n* (%)]	0 (0.0(	2 (8.7)	.495

**Table 7:** Sex and continuous outcomes: univariable analysis.

Gender vs. continuous variables (univariable analysis)	Male (*n* = 18)	Female (*n* = 23)	*P*-value
Operative time [minutes ± SD]	100 ± 29.1	108.2 ± 18.6	.276
EBL [mL ± SD]	47.2 ± 11.8	58.7 ± 28.8	.121
LOS [days ± SD]	6.5 ± 3.7	5.6 ± 1.5	.324

EBL, estimated blood loss; LOS, length of stay.

**Table 8:** Residency status and categorical outcomes: univariable analysis.

Immigrant vs. categorical variables (univariable analysis)	Local (*n* = 32)	Immigrant (*n* = 9)	*P*-value
Asymptomatic [*n* (%)]	7 (21.9)	0 (0.0)	.315
Multiple Organ Involvement [*n* (%)]	3 (9.4)	1 (11.1)	1.0
Post-op complication [*n* (%)]	11 (34.4)	3 (33.3)	.196
Recurrence [*n* (%)]	2 (6.25)	0 (0.0)	.495

**Table 9:** Residency status and continuous outcomes: univariable analysis.

Immigrant vs. continuous variables (univariable analysis)	Local (*n* = 32)	Immigrant (*n* = 9)	*P*-value
Operative time [minutes ± SD]	107.8 ± 24.8	93.3 ± 16.4	.108
EBL [mL ± SD]	54.7 ± 26.5	50 ± 0	.602
LOS [days ± SD]	6.2 ± 3	5.44 ± 1.01	.472

EBL, estimated blood loss; LOS, length of stay.

**Table 10:** Animal exposure and categorical outcomes: univariable analysis.

Contact with dogs or domestic animal vs. Categorical variables (univariable analysis)	Yes (*n* = 31)	No (*n* = 10)	*P*-value
Asymptomatic [*n* (%)]	7 (22.6)	0 (0.0)	.164
Multiple Organ Involvement [*n* (%)]	3 (9.7)	1 (10)	1.0
Post-op complication [*n* (%)]	10 (34.4)	4 (40)	.712
Recurrence [*n* (%)]	2 (6.5)	0 (0.0)	1.0

**Table 11:** Animal exposure and continuous outcomes: univariable analysis.

Contact with dogs or domestic animal vs. Continuous variables (univariable analysis)	Yes (*n* = 31)	No (*n* = 10)	*P*-value
Operative time [minutes ± SD]	104.5 ± 26.1	105 ± 15.8	.956
EBL [mL ± SD]	54.8 ± 26.9	50 ± 0	.577
LOS [days ± SD]	6.06 ± 2.9	5.9 ± 1.73	.869

EBL, estimated blood loss; LOS, length of stay.

## Discussion

In this study, we analyzed the clinical characteristics, diagnostic modalities, and treatment outcomes of pediatric patients with hydatid cyst disease, thereby identifying key patterns in organ involvement, symptomatology, and prognosis. The liver and lungs emerged as the most commonly affected organs, which is consistent with established dissemination patterns of *Echinococcus granulosus* (4–7). Most patients presented with abdominal pain, cough, and fever; however, a notable proportion remained asymptomatic. The majority of cases demonstrated single-organ involvement and a solitary cyst, underscoring a tendency toward localized disease in the pediatric population. Surgical intervention, particularly open deroofing, was the primary treatment modality, and recurrence was infrequent, being observed in only a small number of cases during follow-up (median 12 months). Taken together, these findings highlight the favorable outcomes associated with early surgical management and the generally low risk of recurrence in localized pediatric hydatid disease.

To contextualize our results, we compared our findings with previous studies from Turkey, Tunisia, and Iran, focusing on demographics, clinical manifestations, organ distribution, and imaging modalities. In our study, most affected children were 7–13 years old, with a slight female predominance (56.1%); this is consistent with findings from South Iran (15), where the mean age was 11.5 years and females constituted 56.1% of cases. Likewise, a study from northwest Iran reported a comparable age group (mean 7.9 years) but a slight male predominance (54.2%) (16). Contact with dogs or domestic animals was common among our patients (75%), aligning closely with the South Iran study, which reported a 68.4% contact rate (15). Regarding symptoms, abdominal pain was predominant among our patients, whereas in South Iran, right upper quadrant pain was noted in 100% of liver cyst cases and phlegm was the most frequent symptom in lung involvement (15). Cough, which was also frequently observed in our cohort, was similarly the most common symptom in the northwest Iran study (16). In terms of organ involvement, the liver was the most frequently affected organ in our cohort, followed by the lung, spleen, and kidney. This pattern mirrors the findings of one Turkish study, where the liver was the primary site (*n* = 170) and the lung the second most common (5). However, another Turkish study (4) reported that, in children, lung involvement was more common than liver involvement; a similar trend was reported in Tunisia (7), where 61.8% of cases involved the lung and 34.85% involved the liver. While multi-organ involvement was rare in our cohort (10%), it was similarly low in the South Iran study (15), where only 3.5% of patients had multiple organ cysts.

Regarding imaging, diagnosis in our study was primarily based on CT alone (63.4%) or a combination of CT and ultrasound (36.6%), while follow-up imaging predominantly utilized CT (85.4%).In the Turkish study (4), diagnosis was based on abdominal ultrasound screening and was confirmed with CT and surgery. Similarly, ultrasonography was the primary diagnostic tool in South Iran (15), indicating a consistent reliance on imaging modalities across different regions.

In our study, most hydatid cyst cases were diagnosed by CT alone, with the remainder identified through a combination of CT and ultrasonography (US).While US is frequently the first-line imaging modality in pediatric populations due to its safety, low cost, and accessibility, it has limitations in lesion characterization, particularly when differentiating between cysts, abscesses, and tumors (17, 18). Conventional US demonstrates around 90% sensitivity and specificity for hydatid cysts but lacks the capacity to evaluate enhancement patterns, which can lead to diagnostic ambiguity (17). By contrast, CT provides enhanced lesion characterization and greater than 90% sensitivity through its ability to detect gas content and calcification within cysts, particularly when using multiphasic contrast-enhanced imaging (17–19). These advantages likely contributed to CT being the primary diagnostic tool in our cohort. This aligns with WHO recommendations, which support the use of US as the initial imaging modality, complemented by CT in complex or ambiguous cases (19, 20).

An important finding of our study was the predominant use of CT, either alone or in combination with ultrasonography. However, current WHO and WHO-IWGE guidance emphasizes ultrasonography as the preferred first-line imaging modality for diagnosis and staging in organs accessible to ultrasound, especially for uncomplicated abdominal/hepatic cystic echinococcosis, while CT and MRI serve complementary roles in selected or complex cases. In our cohort, frequent CT use likely reflected referral bias to a pediatric surgical center, the need for preoperative anatomical mapping, pulmonary disease evaluation, multiorgan assessment, and diagnostic uncertainty. This CT-heavy imaging pattern should therefore be interpreted as a feature of retrospective real-world practice rather than an intended recommendation for universal CT-first evaluation in children, and it represents an important limitation of the present study (8–12).

Current WHO guidance generally favors imaging follow-up (“watch and wait”) for uncomplicated inactive CE4–CE5 cysts rather than routine intervention. In our cohort, management reflected retrospective real-world pediatric surgical referral practice rather than a prospectively applied stage-based treatment protocol. Because a dedicated variable for operative indication was not systematically recorded, a uniform case-by-case stage-specific rationale could not be reconstructed for all surgically treated CE4–CE5 lesions (8, 10).

The preferred treatment modality in our study was open deroofing combined with albendazole therapy. Open surgery remains the conventional approach to hydatid cyst (HC) management, particularly in regions with limited access to minimally invasive techniques (19). Nevertheless, surgical intervention alone is associated with a significant risk of recurrence. Albendazole, when used as adjunct therapy, has been shown to reduce cyst viability, shrink cysts, and minimize recurrence risk (19). This combination approach is supported by existing literature, including findings by Gavara et al., who reported that adjunctive albendazole therapy significantly lowers recurrence rates compared to surgery alone (20). In our cohort, the dominant management strategy was open surgery combined with albendazole. Adjunctive albendazole remains an important component of management because it may reduce cyst viability and help lower recurrence risk when used alongside intervention in selected cases. This combined approach is consistent with contemporary evidence supporting multimodal treatment tailored to cyst stage, organ involved, and local expertise (8, 10). These practices are consistent with WHO guidelines, which emphasize a stage-specific, image-guided approach to treatment and endorse the integration of surgery and anti-parasitic therapy where appropriate.

Postoperative complications were relatively common, with respiratory issues such as pneumonia being the most frequently encountered, followed by intra-abdominal collections, pleural complications, air leaks, and readmissions. Less frequently observed complications included biliary leaks and wound infections. These findings are consistent with previous literature, which identifies pulmonary and biliary complications as among the most common postoperative issues in hydatid disease surgery, particularly in cases involving thoracoabdominal involvement or a large cyst burden (21). Despite these challenges, the recurrence rate in our cohort was low over the follow-up period, aligning with studies reporting reduced recurrence when surgical treatment is combined with adjunctive antiparasitic therapy (20, 21). Furthermore, statistical analyses revealed no significant associations between demographic variables—such as age, gender, residency, or animal exposure—and surgical outcomes, suggesting that patient-specific factors may play a limited role in predicting complications or recurrence. Similar conclusions have been drawn in previous studies, which highlight the complexity of hydatid disease outcomes and the importance of intraoperative factors and postoperative management in shaping prognosis (21).

Cystic echinococcosis is a zoonotic disease transmitted from animals to humans. Common modes of transmission include direct contact, indirect contact, transmission through food and water. Thus, community education is important for disease control by raising awareness about risks associated with handling high-risk animals, emphasizing proper hygiene, and ensuring adequate cooking of meat (22). Effective surveillance is essential for monitoring the prevalence of cystic echinococcosis. Surveillance systems should include regular screening of both livestock and humans for *Echinococcus* infection (22).

Although open surgery was the dominant approach in our cohort, minimally invasive treatment warrants mention. In selected pediatric hepatic cases, laparoscopic management has been reported as feasible and safe, with potential advantages including less postoperative pain, earlier ambulation, and shorter hospital stay when careful patient selection is applied. However, its use depends on cyst location, number, accessibility, risk of spillage, and local surgical expertise. No laparoscopic cases were included in our series, so our study does not permit direct comparison between open and minimally invasive approaches (23).

This study has several limitations. First, its retrospective single-center design and recruitment from a pediatric surgical service introduce referral and selection bias toward operable and more complex cases. Second, imaging pathways were not standardized, and CT predominated despite the recognized role of ultrasonography as the preferred first-line modality in organs accessible to ultrasound. Third, WHO-IWGE staging is most directly applicable to ultrasound-characterized lesions, particularly uncomplicated abdominal/hepatic cysts; therefore, retrospective stage assignment in a CT-heavy, multiorgan cohort should be interpreted cautiously. Fourth, serological testing was not performed uniformly and was used as an adjunct rather than a mandatory diagnostic criterion. Fifth, the sample size was small, limiting statistical power and precluding robust multivariable modeling. Finally, the follow-up duration was sufficient to describe early recurrence in most patients but remains limited for assessment of long-term outcomes (8–12). These findings may also help inform future multicenter work under the Ministry of Health to better characterize pediatric cystic echinococcosis across Northern, Central, and Southern Jordan.

In conclusion, hydatid cysts are relatively common among the pediatric population, particularly in children with a history of contact with domestic animals. The combined use of surgical and medical management has been shown in multiple studies to be an effective treatment strategy. To enhance the validity and generalizability of these findings, future research should employ multicenter, prospective study designs with extended follow-up to better assess long-term outcomes and treatment efficacy.

## Conclusion

Open surgery combined with albendazole was associated with low early recurrence and acceptable postoperative morbidity in this single-center pediatric cohort. However, the retrospective design, CT-heavy imaging practice, and limited follow-up warrant cautious interpretation. Future multicenter studies with longer surveillance and more explicit stage-conscious management pathways are needed to better define the roles of surgery, medical therapy, and observation in children with cystic echinococcosis.

## Data Availability

The original contributions presented in the study are included in the article/Supplementary Material, further inquiries can be directed to the corresponding author.
