# Supplementary material for: Clinical profile, imaging characteristics, and operative outcomes of pediatric cystic echinococcosis in Northern Jordan: a single-center retrospective study
Source: Front Surg. 2026 Apr 29;13:1779069. doi: 10.3389/fsurg.2026.1779069 (PMC13168126; doi:10.3389/fsurg.2026.1779069)
Supplement: Supplementary file 1 [file Table1.docx]

**Supplementary Table 1. Organ involvement according to WHO cyst stage, management approach, and documented clinical context in the study cohort**

| **Organ location** | **WHO cyst stage** | **No. of patients** | **Management approach** | **Documented clinical context from retrospective chart review†** |
| --- | --- | --- | --- | --- |
| Kidney | CE3a | 1 | Surgery + albendazole | Operative indication |
| Liver | CE1 | 4 | Surgery + albendazole | Operative indication |
| Liver | CE2 | 2 | Surgery + albendazole | Operative indication |
| Liver | CE3a | 4 | Surgery + albendazole | Operative indication |
| Liver | CE3b | 5 | Surgery + albendazole | Operative indication |
| Liver | CE4 | 4 | Surgery + albendazole | Abdominal pain (1/4), cough (1/4), fever (1/4), NR (1/4) |
| Liver | CE5 | 4 | Surgery + albendazole | Abdominal pain (4/4) |
| Lung | CE1 | 5 | Surgery + albendazole | Operative indication |
| Lung | CE3b | 6 | Surgery + albendazole | Operative indication |
| Lung | CE4 | 3 | Surgery + albendazole | Cough (2/3), shortness of breath (2/3), fever (1/3) |
| Lung | CE5 | 2 | Surgery + albendazole | Fever (1/2), asymptomatic (1/2) |
| Spleen | CE1 | 1 | Surgery + albendazole | Operative indication |
| Spleen | CE2 | 2 | Medical management only (n = 1); Surgery + albendazole (n = 1) | Operative indication |
| Spleen | CE3a | 2 | Surgery + albendazole | Operative indication |
| Spleen | CE3b | 1 | Surgery + albendazole | Operative indication |
| Spleen | CE4 | 1 | Surgery + albendazole | NR |
| † Operative indication was not available for all cases in the retrospective dataset. Therefore, this table presents the documented clinical presentation when available. Patients with multiorgan disease were counted under each affected organ; accordingly, organ-specific totals exceed the total number of patients. **NR**, not reported. | | | | |
